# Physical Activity and Fear Avoidance Over Time in Patients With Acute/Subacute Versus Chronic Unilateral Vestibulopathy: A Prospective Study

**DOI:** 10.1097/MAO.0000000000004704

**Published:** 2025-11-25

**Authors:** Hanna M. Koppelaar-van Eijsden, Lien Van Laer, Constanza Fuentealba Bassaletti, Ann Hallemans, Vincent Van Rompaey, Luc Vereeck, Tjard R. Schermer, Tjasse D. Bruintjes

**Affiliations:** a Apeldoorn Dizziness Centre, Gelre Hospitals, Apeldoorn; b Department of Otorhinolaryngology, Leiden University Medical Center, Leiden, The Netherlands; c Department of Rehabilitation Sciences and Physiotherapy/Movant, Faculty of Medicine and Health Science; d Multidisciplinary Motor Centre Antwerp (M^2^OCEAN), Department of Rehabilitation Sciences and Physiotherapy/Movant, University of Antwerp, Antwerp, Belgium; e Department of Rehabilitation Sciences, Ghent University, Ghent; f Department of Otorhinolaryngology and Head & Neck Surgery, Antwerp University Hospital, Antwerp, Belgium; g Department of Translational Neurosciences, Faculty of Medicine and Health Sciences, University of Antwerp, Antwerp, Belgium; h Department of Primary and Community Care, Radboud Institute for Health Sciences, Radboud University Medical Center, Nijmegen, The Netherlands

**Keywords:** Fear avoidance beliefs, Physical activity, Unilateral vestibulopathy

## Abstract

**Introduction:**

This study investigates how physical activity and fear avoidance beliefs change over time in patients with acute/subacute unilateral vestibulopathy (UVP), compared to those with chronic UVP. It also explores which baseline factors influence activity changes and the relationship between physical activity and fear avoidance beliefs over time.

**Materials and methods:**

In this prospective cohort study, adults with UVP were followed over 10 weeks. Physical activity was measured using accelerometry, and fear avoidance beliefs were assessed via the Vestibular Activities Avoidance Instrument. Subgroup analyses compared acute/subacute and chronic UVP, as well as high- and low-risk groups for persistent complaints. Mixed model analysis identified factors associated with changes in physical activity, and correlations assessed the relationship between activity and fear avoidance beliefs.

**Results:**

Acute/subacute UVP patients increased physical activity significantly over time (+198 min/week), while the chronic group showed no substantial change. Factors such as time since onset, baseline fear avoidance, and etiology influenced activity changes. At baseline, higher fear avoidance was moderately associated with lower activity, though this link weakened at follow-up. Within the acute/subacute group, those at low risk for persistent complaints had consistently higher activity levels than both high-risk and chronic patients.

**Discussion:**

The findings highlight a connection between fear avoidance beliefs and physical activity, particularly in acute/subacute UVP. These insights underscore the importance of evaluating fear avoidance in early stages and suggest a role for psychological factors in designing tailored treatment strategies.

## Introduction

Unilateral vestibulopathy (UVP) is a heterogeneous disorder of the peripheral vestibular system. There are several conditions that can cause UVP, such as vestibular neuritis, Meniere’s disease, labyrinthitis, or unilateral vestibular schwannoma.^1–3^ UVP is characterized by dizziness and/or vertigo, imbalance, and/or oscillopsia.^1^ As a result, the patients experience disrupted spatial orientation and difficulties in maintaining postural stability. Staying physically active represents a critical component in the rehabilitation of patients with UVP, contributing to the improvement of balance, coordination, and overall physical function.

Vestibular rehabilitation (VR) has been shown to facilitate the central compensation process in UVP.^4^ VR is a personalized exercise regime tailored to the individual’s symptoms and may include habituation exercises to decrease dizziness by repeated exposure to stimuli that provoke the symptoms, gaze stabilization exercises to optimize vestibular ocular reflex (VOR) function, and balance training to improve postural stability. It is advised to start VR as soon as possible after UVP is diagnosed. However, it is also effective in a chronic state.^4,5^ Despite the benefits of VR, many patients with UVP experience hesitation or resistance to physical activity, primarily due to fear of triggering their vestibular symptoms (fear avoidance beliefs), which can lead to a vicious cycle of avoidance behavior.^6^

In our previous cross-sectional study, we observed that in patients with acute/subacute UVP, lower levels of physical activity were associated with a higher degree of fear avoidance beliefs, whereas in patients with chronic UVP, fear avoidance beliefs were not associated with physical activity.^7^ However, how physical activity and fear avoidance beliefs evolve over time in patients with acute/subacute UVP remains unclear. Gaining insight into this development is important, as it may significantly impact daily life in patients with vestibular hypofunction.^6^ Dunlap et al^6,8^ demonstrated that increased fear avoidance beliefs were associated with moderate to severe disability, as well as activity limitations and participation restrictions due to dizziness after 3 months.

The primary aim of this study was therefore to assess how physical activity develops over time in patients with acute/subacute UVP, and to compare this change to patients with chronic UVP. Second, we aim to identify which baseline demographic and clinical factors influence the development of physical activity over time and explore how the relationship between physical activity and fear avoidance beliefs evolves over time in patients with acute/subacute and chronic UVP.

## Materials and methods

### Ethical considerations

This study was conducted in accordance with the Declaration of Helsinki and was approved by the ethics committee of the Antwerp University Hospital (21/12/181) and the Leiden Den Haag Delft ethics committee (NL 77986.058.21). All study subjects gave written informed consent before participating in the study.

### Design

We conducted a prospective cohort study based on a convenience patient sample without prior sample size calculation. The data were collected between May 2021 and November 2024. The sample was composed of 2 separate studies in patients with UVP, one study performed at the tertiary care dizziness center of Gelre Hospitals in Apeldoorn, the Netherlands. The other study was conducted at 3 hospitals in Belgium (Antwerp University Hospital, Jessa Hospital, Hasselt, and Sint-Lievenspoort Rehabilitation Center, Ghent).

We included adult individuals with a UVP if they met the Bárány Society diagnostic criteria for UVP, which concerns complaints of dizziness and/or balance problems and vestibular function loss as confirmed by the vHIT and/or caloric testing.^1^ In addition to a baseline measurement, we performed a follow-up measurement at 10 weeks. Therefore, to be included in the current study, complete data for the measurements of physical activity and fear avoidance beliefs at baseline and follow-up were required. Patients who dropped out (n=28) of the initial cohort were replaced with newly available participants who met the extended set of inclusion criteria.^7^

At baseline, all participants were advised by a medical doctor or the researcher to engage in as much physical activity as possible, as this is considered standard care following UVP.^4^ In addition, since VR is highly recommended for individuals with UVP, it was made available to all participants either through a home exercise program or by referral to a primary care physical therapist with expertise in vestibular rehabilitation. The VR exercises included gaze stability, balance, and habituation exercises, tailored to the patient’s specific complaints. There were no differences in the exercises provided to the acute/subacute and chronic groups.

At baseline, participants were classified into 2 subgroups: acute/subacute or chronic. The acute/subacute phase was defined as inclusion within the first three months following the onset of vestibular symptoms. The chronic phase was classified as symptom onset 3 months or more before inclusion.^4^

### Primary outcome measures

Physical activity levels were quantified using MOX1 loggers (Maastricht Instruments). The MOX1 is a waterproof tri-axial accelerometer that is worn on the upper leg.^9^ Study participants wore the MOX1 logger for 1 week at baseline and 1 week at follow-up (with a minimum of 3 days). If measurements were made for more or less than 7 days, the data were interpolated or extrapolated to 1 week. The analysis was restricted to waking hours, operationalized as the period between wake-up time and bedtime.^7^ Time spent sitting/lying, standing, performing low physical activity (LPA), moderate physical activity (MPA), and vigorous physical activity (VPA) was calculated by the device’s software (IDEEQ. 2.0) and expressed as min/week. Total physical activity (TPA) was defined as the sum of LPA, MPA, and VPA and used as the primary outcome measure for the analysis.

Fear avoidance beliefs were objectified using the total score of the Dutch 9-item version of the Vestibular Activities Avoidance Instrument (VAAI) at baseline and follow-up.^10,11^ The 9 items cover work, fear, and activity and participation and are scored on a 7-point Likert scale. The score ranges from 0 to 54 points. The higher the score, the higher the likelihood of the presence of fear avoidance beliefs. In individuals with vestibular disorders, the VAAI has demonstrated excellent internal consistency (Cronbach α=0.91) and test-retest reliability (ICC=0.92), and the minimal detectable change (MDC) is 8.9 points.^8,11^ A VAAI score ≥26 points identifies individuals at risk of persistent moderate to severe disability due to dizziness.^8^

### Secondary outcome measures

The perceived handicap due to dizziness as a representation of quality of life was measured by means of the Dizziness Handicap Inventory (DHI) at baseline.^12^ The DHI consists of 25 items, with the total score ranging from 0 to 100 points and a higher score indicating greater dysfunction.^13,14^ The Dutch language version of the DHI (DHI-DLV) has been shown to be a reliable instrument with sufficient construct validity in individuals with vestibular disorders.^15^ The severity of dizziness is classified as mild (DHI score 0 to 30 points), moderate (DHI score 31 to 60 points), and severe (DHI score 61 to 100 points).^14^ An improvement of more than 11 points can be considered clinically relevant.^15,16^

### Baseline characteristics and predictors

Predictors were only collected at baseline to investigate their association with the change in TPA from baseline to follow-up.

Vestibular function was tested by means of the horizontal video-Head Impulse Test (vHIT) and/or caloric testing. The vHIT (ICS-Impulse vHIT, Otometrics/Natus) measures the VOR and is expressed by the VOR gain. A horizontal VOR gain lower than 0.7 or a VOR gain side difference of >0.30 was considered abnormal. If needed in the diagnostic process, caloric testing was done in Gelre Hospitals by using Vestlab (Otometrics), at Antwerp University Hospital using Kaloristar (Biomed), and at Jessa Hospital and Sint-Lievenpoort Rehabilitation Center using Aquastar (Difra, Belgium). A caloric side difference of 24% or more was classified as abnormal.

Data on the primary UVP diagnoses and etiology, time since onset (TSO) of the UVP, age, and sex obtained from the patient history were also collected. The etiology was divided into 2 groups, that is, inflammatory (e.g. vestibular neuritis) versus noninflammatory (e.g. resection of a vestibular schwannoma).

### Statistical analysis

Baseline characteristics were described using frequencies and percentages or means and SD when appropriate, unless otherwise indicated. For continuous variables, a normality check was done by means of visual inspection and statistical tests (namely, Kolmogorov-Smirnov and Shapiro-Wilk). Differences in baseline demographic and clinical characteristics between the acute/subacute and chronic subgroups were assessed using χ^2^ or an independent samples *t* test. Regarding the questionnaires, differences between the subgroups were assessed by means of an independent samples *t* test and differences within the subgroups using the paired *t* test. Effect sizes were expressed using Cohen’s *d*. Parametric tests were chosen when the subgroups were sufficiently large, that is, n≥25, otherwise the nonparametric variant was chosen.^17^

We used mixed-model analysis (MMA) to longitudinally analyze associations between the time since onset at baseline (ie, the acute/subacute UVP vs. the chronic UVP subgroup) as the independent variable and TPA as the dependent variable, with the model including a random intercept but not a random slope. Time since onset (acute/subacute vs. chronic UVP) was also entered into the model as an interaction term with time (ie, baseline of follow-up measurement). We applied the generally accepted rule of thumb for the maximum number of covariates to be entered in a multivariable analysis, viz., 1 covariate was added to the model for every 10 patients.^18^ The following covariates and predictors were also entered in the full MMA model: baseline fear avoidance beliefs (ie, VAAI score), age (in years), ipsilesional vestibular hypofunction (vHIT gain), etiology (inflammatory or noninflammatory), and sex (male or female).

To explore the relationship between TPA and fear avoidance beliefs, we calculated Spearman correlation coefficients for the correlation between TPA and VAAI scores at baseline, follow-up, and change in scores. Furthermore, we explored differences in TPA between patients with a high risk of persistent disability due to dizziness (ie, having a VAAI score ≥26 points at baseline) and patients with a low risk of persistent disability (ie, having a VAAI score <26 points at baseline).^8^ Patients classified as “high risk” exhibited markedly elevated levels of fear avoidance behavior, in contrast to those in the low-risk category.

Data were analyzed using IBM Statistics SPSS for Windows version 28. A *P* value <0.05 was considered statistically significant.

## Results

### Patient characteristics

A total of 88 patients with UVP were included, equally distributed between the acute/subacute and chronic subgroups. Table 1 presents the patient characteristics for both subgroups. At baseline, the acute/subacute subgroup was younger (52 vs. 62 y), and presented with a lower mean gain on the horizontal vHIT (0.55 vs. 0.72), a higher percentage of caloric asymmetry (71.5% vs. 48.2%), a higher percentage of iatrogenic etiologies, and a lower percentage of idiopathic peripheral causes compared with the chronic subgroup.

**Table 1:** Baseline characteristics for participants with acute/subacute UVP and chronic UVP

	Acute/Subacute Subgroup (n=44)	Chronic Subgroup (n=44)	*P* for Difference Between Subgroups
Age, mean (SD), y	52.6 (14.8)	62.2 (15.0)	**0.003^a^**
Age range, y	22-78	21-83	
Females, n (%)	18 (40.9)	21 (47.7)	0.520^b^
Primary diagnosis, n (%)			**<0.001^c^**
Vestibular neuritis	19 (43.2)	18 (40.9)	
Resection vestibular schwannoma	15 (34.1)	0	
Idiopathic peripheral	0	13 (29.5)	
Meniere’s disease	0	7 (15.9)	
Labyrinthitis	4 (9.1)	6 (13.6)	
Other iatrogenic	3 (6.8)	0	
Gentamicin injection	2 (4.5)	0	
Traumatic	1 (2.3)	0	
Etiology, n (%)			
Noninflammatory	21 (47.7)	20 (45.5)	0.831^b^
Inflammatory^d^	23 (52.3)	24 (54.5)	
DHI			
Mean (SD)	39.7 (18.6)	50.5 (17.0)	
Vestibular function			
VOR gain ipsilesional^e^			**<0.001^a^**
Mean (SD)	0.55 (0.21)	0.72 (0.23)	
Range	0.21-1.11	0.25-1.11	
VOR gain contralesional^e^			0.071^a^
Mean (SD)	0.97 (0.22)	0.90 (0.14)	
Range	0.67-1.8^f^	0.62-1.27^f^	
Caloric asymmetry^g^			**<0.001^a^**
Mean % (SD)	71.5 (21.6)	48.2 (16.8)	
Range %	33-100	15-88	
Affected side, n (%) left	21 (47.7)	25 (56.8)	0.393^b^

Bold front type was used to indicate a statistically significant difference between acute/subacute and chronic participants.

DHI indicates dizziness handicap inventory; n, number; UPV, unilateral vestibulopathy; VOR, vestibulo-ocular reflex

^a^ From an independent samples *t* test.

^b^ From the χ^2^ test.

^c^ From the Fisher exact test.

^d^ Inflammatory etiology consists of vestibular neuritis and labyrinthitis.

^e^ Missing in 1 acute/subacute and 1 chronic patient.

^f^ In n=5, VOR gains were >1.20, probably caused by slippage or could be an effect of camera placement.

^g^ Missing in 33 acute/subacute and 13 chronic patients; test not administered.

#### Dizziness severity

Based on the DHI, at baseline, 27.3% of the total group experienced mild complaints, 48.9% moderate complaints, and 23.9% had severe complaints.

In the acute/subacute group, a significant and clinically relevant reduction in DHI scores was observed, decreasing from 39.7 ± 18.6 at baseline to 23.0 ± 17.4 at follow-up (*P* < 0.001); overall, symptoms decreased from moderate to mild. The chronic group showed a significant improvement, but not clinically relevant, with scores declining from 50.5 ± 20.0 to 41.7 ± 20.6 (*P* < 0.001); overall, severity of symptoms remained moderate.

#### Measurement parameters

The follow-up time was shorter in the acute/subacute subgroup compared with the chronic subgroup, 65 days (SD 17) versus 95 days (SD 18), respectively (*P*<0.001).

There was no statistically significant difference in the duration of time the MOX1 logger was worn between baseline and follow-up for the total group (mean of 6.6 days at both measurement times, *P*=0.618).

### Level of physical activity over time

**Figure 1:**
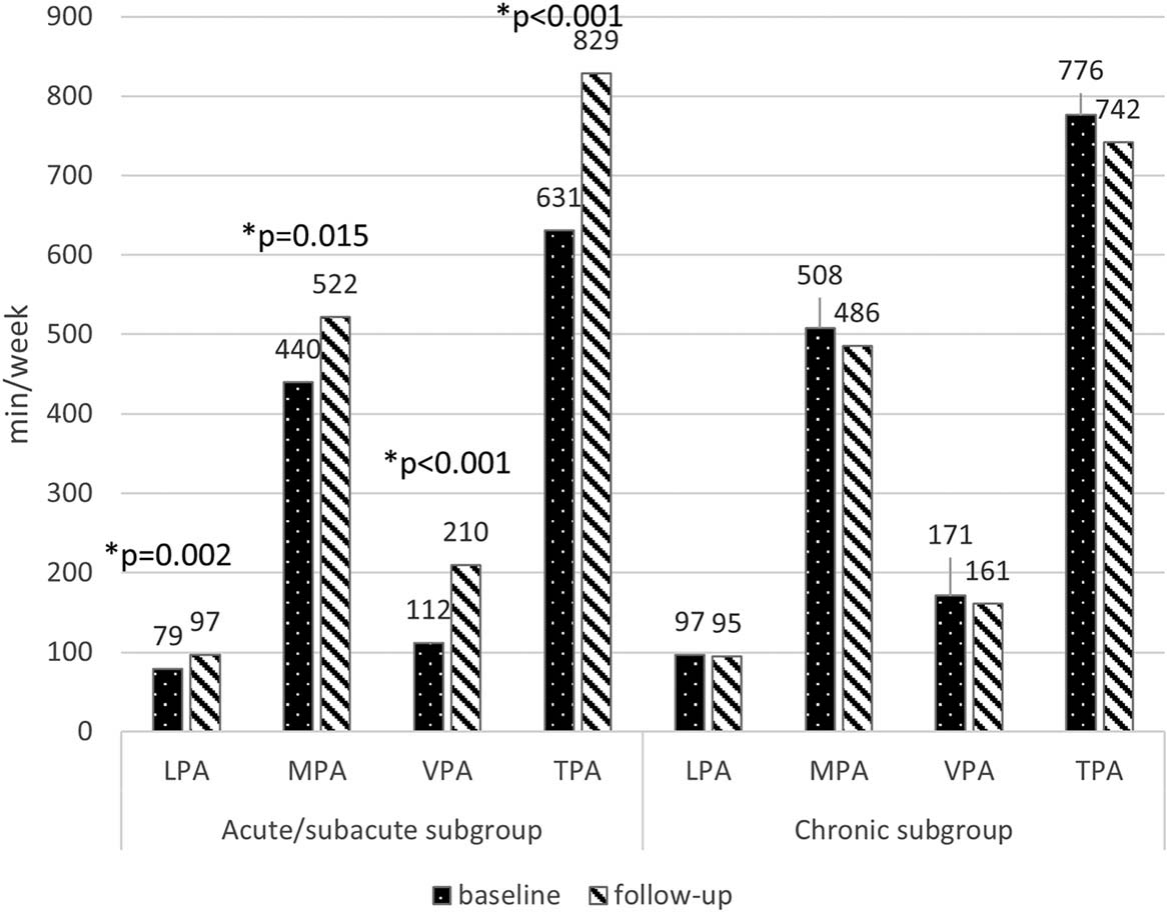
Level of physical activity at baseline and follow-up for the acute/subacute UVP and chronic UVP subgroups. LPA indicates low physical activity; MPA, moderate physical activity; TPA, total physical activity; VPA, vigorous physical activity. *Statistically significant difference within the subgroup (paired *t* test).

**Figure 2:**
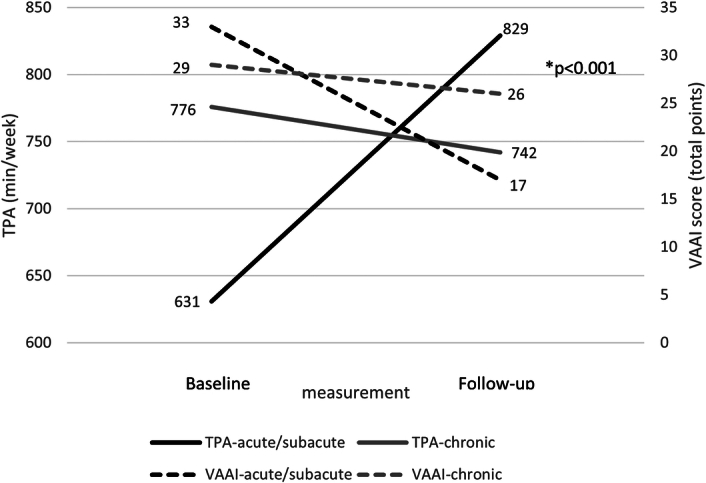
Change in total physical activity (TPA) and Vestibular Activities Avoidance Instrument (VAAI) score from baseline to follow-up in the acute/subacute and chronic UVP subgroups. TPA, total physical activity (solid line, scale on left *y*-axis); VAAI, vestibular activity avoidance instrument (dashed line, scale on right *y*-axis). *Difference in the change in TPA between the acute/subacute and chronic subgroups was tested using an interaction term measurement × subgroup in the mixed model.

**Figure 3:**
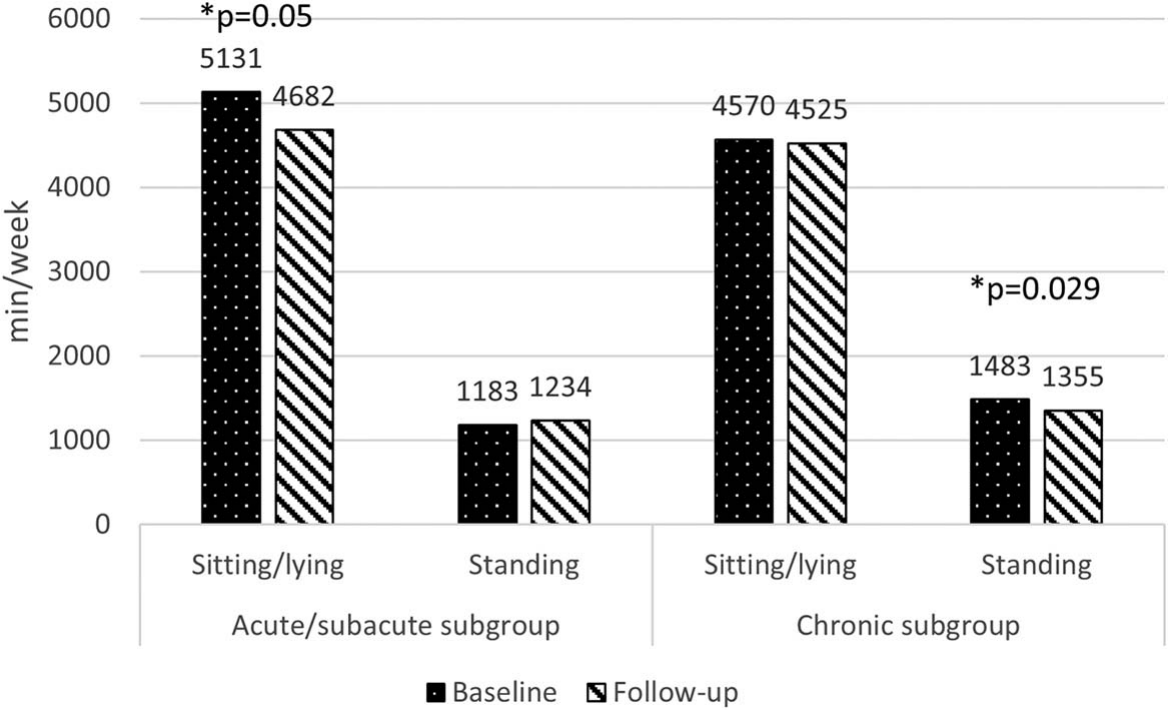
Sedentary behavior in acute/subacute UVP and chronic UVP subgroups. *Statistically significant difference within the subgroup (paired *t* test).

The acute/subacute patients showed a mean increase in TPA of 198 min/week (SD 374), compared with a mean decrease of 34 min/week (SD 170) in the chronic subgroup (Figs. 1 and 2). The observed improvement of physical activity in the acute/subacute subgroup was primarily due to an increase in moderate and vigorous activities and a decrease in sedentary behavior (Figs. 1 and 3). The patients in the chronic subgroup remained relatively stable across all domains of physical activity and sedentary behavior (Figs. 1 and 3).

### Predictors for physical activity at follow-up

The MMA showed the time since onset (acute/subacute versus chronic) × time interaction term to be statistically significant (*P*<0.001, Fig. 2). Baseline fear avoidance beliefs (*P*=0.006) and etiology (*P*=0.009) were significantly associated with the change in TPA, whereas age (*P*=0.145), ipsilesional vestibular hypofunction (vHIT gain) (*P*=0.786), and sex (*P*=0.811) were not.

### Relation between physical activity and fear avoidance beliefs over time

Figure 2 shows the VAAI scores over time, from baseline to follow-up. A greater improvement, indicated by a decrease of 16 points in VAAI total score, was observed in the acute/subacute subgroup, compared with a decrease of 3 points in the chronic subgroup (*P*<0.001, Cohen’s *d*=−1.293).

For the acute/subacute and chronic subgroups combined, the correlation between TPA and VAAI score was moderate at baseline (ρ=−0.439; *P*<0.001), and weak at follow-up (ρ=−0.231; *P*=0.030). In the subgroups, at baseline, a moderate inverse correlation was observed (ρ=−0.512; *P*<0.001) for the acute/subacute subgroup and a weak inverse correlation (ρ=−0.306; *P*=0.043) for the chronic subgroup. At follow-up, the correlations were nonsignificant, namely ρ=−0.255 (*P*=0.095) for the acute/subacute subgroup and ρ=−0.173 (*P*=0.261) for the chronic subgroup.

At baseline, 77.3% of the patients in the acute/subacute subgroup were classified in the high-risk VAAI category, compared with 70.5% of the patients in the chronic subgroup. Table 2 presents the physical activity levels and the VAAI scores of patients in the total group, acute/subacute, and chronic UVP subgroups, categorized by high and low VAAI risk, at both baseline and follow-up.

**Table 2:** Total physical activity (TPA) in high-level and low-level fear avoidance categories

	Baseline VAAI Score Category		
	Low-risk^a^	High-risk^a^	*P* for Difference Between Low- and High-risk Category^b^
Total UVP group			
n (%) in category	23 (26.1)	65 (73.9)	
VAAI score—baseline	17.3 (SD 6.1)	36.1 (SD 6.8)	**<0.001**
VAAI score—follow-up	13.3 (SD 10.3)	24.5 (SD 10.9)	**<0.001**
VAAI score—change	−3.4 (SD 7.7)^c^	−11.5 (SD 12.6)^d^	**0.011**
TPA at baseline	849 (SD 358)	651 (SD 286)	**0.023**
TPA at follow-up	849 (SD 250)	762 (SD 269)	0.125
Change TPA during follow-up	0 (SD 323)^e^	111 (SD 304)^f^	0.153
Acute/subacute UVP group			
n (%) in category	10 (22.7)	34 (77.3)	
VAAI score—baseline	18.0 (SD 5.1)	37.6 (SD 7.7)	**<0.001**
VAAI score—follow-up	11.4 (SD 12.4)	18.8 (SD 9.3)	0.085
VAAI score—change	−6.6 (SD 9.5)^g^	−18.8 (SD 11.5)^d^	0.005
TPA at baseline	917 (SD 490)	546 (SD 261)	**0.024**
TPA at follow-up	983 (SD 243)	783 (SD 303)	**0.035**
Change TPA during follow-up	66 (SD 455)^e^	237 (SD 344)^d^	0.186
Chronic UVP group			
n (%) in category	13 (29.5)	31 (70.5)	
VAAI score—baseline	16.7 (SD 6.9)	34.4 (SD 5.4)	**<0.001**
VAAI score—follow-up	14.8 (SD 8.6)	30.8 (SD 8.8)	**<0.001**
VAAI score—change	−1.9 (SD 5.4)^e^	−3.5 (SD 8.1)^h^	0.561
TPA at baseline	797 (SD 219)	767 (SD 271)	0.598
TPA at follow-up	746 (SD 210)	739 (SD 228)	0.928
Change TPA during follow-up	−51 (SD 171)^e^	−27 (SD 172)^e^	0.908

Bold front type was used to indicate a statistically significant difference.

n indicates number; TPA, total physical activity in min/week; UVP, unilateral vestibulopathy; VAAI, Vestibular Activities Avoidance Instrument.

^a^ Low-risk category, Baseline VAAI score <26 points, that is, low fear avoidance beliefs; High-risk group, Baseline VAAI score ≥26 points, that is, high fear avoidance beliefs.

^b^ From the Mann-Whitney *U* test.

^c^ From the Wilcoxon Signed Rank test, *P*=0.043.

^d^ From the Wilcoxon Signed Rank test, *P*<0.001.

^e^ From the Wilcoxon Signed Rank test, not significant.

^f^ From the Wilcoxon Signed Rank test, *P*=0.016.

^g^ From the Wilcoxon Signed Rank test, *P*=0.047.

^h^ From the Wilcoxon Signed Rank test, *P*=0.010.

In the total group, a statistically significant difference of 198 min/week (*P*=0.023) in favor of the low-risk VAAI category was found between the high-risk and low-risk VAAI categories at baseline. However, the TPA at follow-up and the changes from baseline to follow-up did not differ significantly. In contrast, the increase in physical activity in the high-risk category of 111 min/week was statistically significant (*P*=0.016).

In the acute/subacute subgroup, a significant difference of 371 min/week (*P*=0.024) was observed at baseline in favor of the low-risk VAAI category. At follow-up, the difference between the low-risk and high-risk categories decreased to 200 min/week; however, this difference remained statistically significant (*P*=0.035). The increase in physical activity in the high-risk category of the acute/subacute group of 237 min/week was statistically significant (*P*<0.001).

In contrast to the total group and the acute/subacute subgroup, no statistically significant difference in TPA was observed between the high-risk and low-risk categories in the chronic subgroup.

## Discussion

### Summary of the results

The main goal of this study was to examine the level of physical activity over time in patients with UVP and to compare this trajectory between patients in the acute/subacute phase and patients in the chronic phase. Subsequently, we wanted to identify which patient characteristics and predictors influence total physical activity over time and to explore how the relationship between physical activity and fear avoidance beliefs progresses. Our findings revealed different patterns between the acute/subacute and chronic subgroups during the 2- to 3-month period following baseline. The acute/subacute patients increased their physical activity levels by ~200 minutes per week (or ~30 min per day). This increase was primarily driven by reduced sedentary behavior and greater engagement in moderate to vigorous physical activity (Figs. 1 and 3). In contrast, the chronic patients showed no improvement, maintaining similar activity levels from baseline to follow-up. At follow-up, the acute/subacute subgroup surpassed the chronic subgroup in physical activity levels, suggesting a recovery to normal activity. One possible explanation for the greater improvement in the acute/subacute subgroup is the lower level of baseline activity in the acute/subacute subgroup in comparison with the chronic subgroup. Indeed, symptom severity is typically more pronounced in the first days to weeks after onset,^2,19^ which may contribute to reduced physical activity. In addition, because the follow-up period fell within the acute/subacute phase, this subgroup had greater potential for recovery, as the natural recovery process of UVP was still ongoing.^20^ Factors influencing the level of physical activity over time were baseline VAAI scores and UVP etiology. The acute/subacute subgroup had a higher proportion of medically induced UVPs (noninflammatory), likely resulting in hospitalization and other complications that initially reduced their physical activity.^21,22^ The acute/subacute subgroup was significantly younger, which may have also positively impacted their recovery. However, in the mixed-model analysis, no significant effect of age on the change in physical activity between baseline and follow-up was found, consistent with findings from previous studies.^23^ Other variables, such as vestibular function and sex, were also examined in our analysis, but neither had a significant impact on the change in physical activity. The relationship between vestibular function and physical activity has been explored previously, with one study reporting significant associations between vestibular function—measured by VOR gain—and movement energy expenditure.^24^ However, differences in physical activity measurement methods make direct comparisons with our findings challenging. Another study identified cross-sectional associations between physical activity and postural stability^25^; unfortunately, we did not assess balance performance in our participants. To our knowledge, no other studies have examined the progression of physical activity over time in individuals with vestibular disorders. Therefore, further research is needed to validate our results.

In addition, we aimed to examine the relationship between physical activity and fear avoidance beliefs over time. The correlation between physical activity and fear avoidance beliefs was strongest at baseline but weakened at follow-up. The acute/subacute subgroup showed a stronger relationship at both time points than the chronic subgroup.

Furthermore, when using the VAAI cutoff point of 26 points, as suggested by Dunlap et al,^8^ more distinct patterns emerged. Over 70% of the patients were initially in the high-risk category. Within the acute/subacute group, high-risk patients consistently exhibited lower physical activity levels than low-risk patients at both baseline and follow-up. In contrast, in the chronic subgroup, no differences in physical activity were observed between the high-risk and low-risk categories, either at baseline or during follow-up. Notably, in the high-risk subgroup of the acute/subacute group, TPA improved from 546 minutes at baseline to 783 minutes at follow-up, yet remained well below the 983 minutes observed in the low-risk subgroup at follow-up and was almost equivalent to the level of the chronic subgroups.

The VAAI scores in the high-risk acute/subacute subgroup at follow-up are below the cutoff point, but still higher than those in the low-risk group (18.8 vs. 11.4 points). In the high-risk chronic subgroup, VAAI scores remain elevated and above the cutoff point (30.8 points).

These findings suggest that the distinction between high- and low-risk patients based on the VAAI is particularly relevant in the early phase of UVP. The fact that high VAAI scores in the acute/subacute phase are associated with persistently lower physical activity levels at follow-up—even when some improvement occurs—may indicate that early identification and intervention in these high-risk individuals is crucial. Timely intervention could help prevent the development of long-term fear avoidance behavior and physical inactivity, which are more difficult to reverse in the chronic stage. Therefore, the use of the VAAI in the acute/subacute phase may not only help identify patients at risk of poor recovery but also guide targeted early interventions aimed at improving long-term functional outcomes.

In our study, on average, both the acute/subacute and chronic subgroups met the guidelines for time spent on moderate (150 to 300 min/week) and vigorous (75 to 150 min/week) physical activity set by the World Health Organization (WHO) (Fig. 1).^26^ However, at follow-up, there is still a substantial difference (>200 min/week) regarding the minutes of physical activity/week between the patients in the low-risk acute/subacute subgroup (983 min/week) and the high-risk acute/subacute subgroup (783 min/week), the low-risk chronic subgroup (746 min/week) and high-risk chronic subgroup (739 min/week).

Our results indicate that there are links between fear avoidance beliefs and objectively measured physical activity (PA). Further research into the relationship between objectively measured PA and subjective questionnaires [ie, DHI, vestibular disorders activities of daily living scale (VADL), vestibular activities, and participation questionnaire (VAP)] is warranted to better understand the relationship between activity and symptoms, and to potentially explain the observed differences. Moreover, Herdman et al^27^ showed that self-reported dizziness and handicap were associated with psychological factors such as anxiety, depression, illness perception, cognitive and behavioral responses; therefore, these factors should also be considered as potential predictors for chronic complaints and lower levels of physical activity and taken into account in future research and treatment modalities.

### Strengths and limitations

This study has several strengths, including its multicenter and prospective design, a carefully selected patient group consisting solely of individuals with unilateral vestibular hypofunction (creating a homogeneous group despite varying causes of dysfunction), and the objective measurement of physical activity using an accelerometer.

However, this study also has some limitations. First, the acute/subacute and chronic subgroups exhibited some baseline differences in terms of age, etiology, and vestibular function. However, these factors were accounted for in the analyses. Another limitation was the difference in time between measurements, as the acute/subacute subgroup had a shorter follow-up period than the chronic subgroup, which may potentially lead to an underestimation of the results to the disadvantage of the acute/subacute subgroup. If the acute/subacute subgroup had been followed for the same duration, their recovery might have been even more pronounced, thereby further increasing the difference with the chronic subgroup. Despite the shorter follow-up, the acute/subacute subgroup showed the most substantial changes in physical activity levels, as well as in secondary outcome measures such as the VAAI and DHI.

Another limitation of this study is the lack of data on adherence to the vestibular rehabilitation home exercise program or physical therapy referrals, preventing us from assessing their impact on physical activity levels. Although participants were encouraged to stay active and received a home exercise program or physical therapy referral, adherence was not measured. This approach appeared to increase activity in the acute/subacute subgroup but was insufficient for the chronic subgroup. A more structured follow-up to monitor adherence could be beneficial in future studies. Moreover, due to the observational design of the study, there was no standardized rehabilitation protocol, and the participants received “usual care,” meaning that the exercise program was individualized based on the patient’s symptoms, resulting in variability in the type and intensity of interventions.

## Conclusions

Patients with acute/subacute UVP demonstrate a marked improvement in physical activity over the course of several months of follow-up, whereas those with chronic UVP show no significant change. Changes in physical activity are influenced not only by time since UVP onset but also by etiology and fear avoidance beliefs. In clinical practice, fear avoidance beliefs appear to be associated with physical (in)activity, particularly during the acute/subacute phase following UVP, and not in the chronic phase.

## Funding

This research was funded by a grant from the University of Antwerp (nr. 42186) and Gelre Hospitals.

## Acknowledgments

The authors thank all the participants for their time and willingness to participate. They also thank all referring doctors from the Antwerp University Hospital, Gelre Hospital, the Jessa Hospital (Hasselt, Belgium), the Rehabilitation Center Sint-Lievenspoort (Ghent, Belgium), and the AZ Turnhout Hospital (Turnhout, Belgium) for their help in the recruitment of participants and their guidance throughout our research project.
